# Acknowledgments

**DOI:** 10.4269/ajtmh.1113siii

**Published:** 2024-09-03

The authors wish to express their deepest gratitude to Carter Center CEO Paige Alexander, Vice President of Health Programs Kashef Ijaz, and staff in Atlanta and across all Carter Center health program-supported countries for their leadership and tireless efforts. Thanks go out to the program directors, Adam Weiss (Guinea worm), Gregory Noland (river blindness, lymphatic filariasis, schistosomiasis, malaria), Kelly Callahan (trachoma), and Eve Byrd (mental health); and country representatives, Emmanuel Miri (Nigeria), Zerihun Tadesse (Ethiopia), Mauricio Sauerbrey (Director, OEPA), Jim Niquette (South Sudan), Sadi Moussa (Mali), Edridah Muheki (Uganda), Sara Lavinia (Sudan), Luccene Desir (Haiti, Dominican Republic), Boukari Gambo (Niger), Abdallah Meftuh (Chad), and Benedict Dossen (Liberia). We extend our thanks to the volunteers, health workers, supervisors, district and regional staff , and Ministry of Health and other government partners whose efforts largely contributed to the achievements documented in this supplement.

Special appreciation is given to Frank Richards, Donald Hopkins, Scott Nash, Obiora Eneanya, Jenna Coalson, Maryann Delea, Samhita Kumar, Anyess Travers, Ursula Kajani, and Emily Staub for their exceptional oversight, coordination, and critical review for the development of this supplement.

Special thanks to guest editor Mark Eberhard, for his diligent oversight of the peer-review process and his remarkable contributions to global health throughout his career.

We would also like to acknowledge the AJTMH editorial staff for making this supplement possible, especially Editor-in-Chief Phil Rosenthal, Managing Editor and Publisher Alison Jaeb, Editorial Production Manager Annie Calacci, and Peer-Review Coordinator Lori Young. Since the inception of the Carter Center’s health programs, more than 100 peer-reviewed articles, many of them documenting historic accomplishments, innovative solutions, and decision-making data, have been published in the *American Journal of Tropical Medicine and Hygiene* (AJTMH), the official journal of the American Society of Tropical Medicine and Hygiene (ASTMH).

For nearly four decades, Carter Center experts have been proud and active members of the ASTMH, and we are honored by the opportunities available to showcase our efforts during the society’s annual meetings. We thank the ASTMH, its leadership, and current and past society presidents for their partnership, including the remarkable tribute to President and Mrs. Carter during the 2023 ASTMH Annual Meeting in Chicago.

We sincerely thank the many donors and partners whose support has been instrumental in achieving the accomplishments of the Carter Center’s Health Programs highlighted in this supplement. Finally, we would like to extend our gratitude to our founders, President and Mrs. Carter, for their vision, passion, direction, and unwavering commitment to alleviating human suffering and making the world a better place.

